# Genomic analysis of *Leishmania turanica* strains from different regions of Central Asia

**DOI:** 10.1371/journal.pntd.0011145

**Published:** 2023-03-06

**Authors:** Tatiana S. Novozhilova, Daniil S. Chistyakov, Lyudmila V. Akhmadishina, Alexander N. Lukashev, Evgeny S. Gerasimov, Vyacheslav Yurchenko

**Affiliations:** 1 Faculty of Biology, M.V. Lomonosov Moscow State University, Moscow, Russia; 2 Martsinovsky Institute of Medical Parasitology, Sechenov University, Moscow, Russia; 3 Faculty of Bioengineering and Bioinformatics, Lomonosov Moscow State University, Moscow, Russia; 4 Life Science Research Centre, Faculty of Science, University of Ostrava, Ostrava, Czech Republic; Charité University Medicine Berlin, GERMANY

## Abstract

The evolution in *Leishmania* is governed by the opposite forces of clonality and sexual reproduction, with vicariance being an important factor. As such, *Leishmania* spp. populations may be monospecific or mixed. *Leishmania turanica* in Central Asia is a good model to compare these two types. In most areas, populations of *L*. *turanica* are mixed with *L*. *gerbilli* and *L*. *major*. Notably, co-infection with *L*. *turanica* in great gerbils helps *L*. *major* to withstand a break in the transmission cycle. Conversely, the populations of *L*. *turanica* in Mongolia are monospecific and geographically isolated. In this work, we compare genomes of several well-characterized strains of *L*. *turanica* originated from monospecific and mixed populations in Central Asia in order to shed light on genetic factors, which may drive evolution of these parasites in different settings. Our results illustrate that evolutionary differences between mixed and monospecific populations of *L*. *turanica* are not dramatic. On the level of large-scale genomic rearrangements, we confirmed that different genomic loci and different types of rearrangements may differentiate strains originated from mixed and monospecific populations, with genome translocations being the most prominent example. Our data suggests that *L*. *turanica* has a significantly higher level of chromosomal copy number variation between the strains compared to its sister species *L*. *major* with only one supernumerary chromosome. This suggests that *L*. *turanica* (in contrast to *L*. *major*) is in the active phase of evolutionary adaptation.

## Author summary

Leishmaniasis is one of the major neglected tropical diseases caused by members of the genus *Leishmania*. It can manifest in the symptoms ranging from rapidly self-healing ulcers in the cases of cutaneous leishmaniasis (CL) to systemic multiorgan failures in the cases of visceral leishmaniasis. The CL can be of anthroponotic (ACL, mainly caused by *L*. *tropica*) and zoonotic (ZCL, mainly caused by *L*. *major*) origin. In Central Asia, great gerbils are the main animal reservoirs for ZCL. These animals may be co-infected with *L*. *major*, *L*. *turanica*, and *L*. *gerbilli*. The evolution in *Leishmania* is driven by clonality and genetic exchange, with geographical isolation playing an important role. In this sense, *Leishmania* spp. may either co-exist in the same area (mixed infections) or be geographically isolated without mixing-up with other species (monospecific infections). *Leishmania turanica* in Central Asia presents a good model to compare these two modes. Indeed, in most areas of great gerbils’ distribution, populations of *Leishmania* spp. are mixed. Conversely, the populations of *L*. *turanica* in Mongolia are monospecific and geographically isolated. In this work, we compare genomes of several strains of *L*. *turanica* in order to shed light on genetic factors, which may drive evolution of these important parasites.

## Introduction

Leishmaniasis is one of the major neglected tropical diseases in almost 100 countries. Its etiological agents are *Leishmania* spp. of the family Trypanosomatidae (Euglenozoa: Kinetoplastea) [1,2]. Well over 10 million people are infected worldwide with over one and a half million new cases being reported annually and 350 million people being at risk of infection [3]. Clinical manifestations of the disease range from often self-healing cutaneous ulcers in the cases of cutaneous leishmaniasis (CL) to systemic multiorgan failures in the cases of visceral leishmaniasis (VL) [4,5]. The CL can be of anthroponotic (ACL) and zoonotic (ZCL) origin mainly caused by *L*. *tropica* and *L*. *major*, respectively [6,7].

In Central Asia, great gerbils (*Rhombomys opimus*) serve as the main animal reservoirs for ZCL, although other animal species may also be involved [8–10]. These animals may be infected with three *Leishmania* spp.: i) *L*. *major* (pathogenic to humans, causing CL), ii) *L*. *turanica* (presumably, gerbil-restricted but causing mild self-healing symptoms in humans in experimental infections), and iii) *L*. *gerbilli* (strictly gerbil-restricted) [11–14]. In some instances, all three species may infect the same animal [15,16]. The main vectors in this area are sand flies *Phlebotomus papatasi*, *P*. *caucasicus*, *P*. *andrejevi*, *P*. *alexandri*, and *P*. *mongolensis* [10].

The human ZCL, which is caused by *L*. *major* in these areas, invariably happens in the context of *L*. *turanica* or, rarely, *L*. *gerbilli* infection. Importantly, co-infecting agents play an important functional role. For example, *L*. *turanica* was shown to aid *L*. *major* survival in gerbils during the 6–10 months gap in the transmission cycle of these flagellates [12]. The co-infections involving *L*. *turanica* and *L*. *major* in great gerbils are evolutionarily beneficial over the single-species infections. In the experimental settings, monospecific infections with *L*. *major* and *L*. *turanica* persisted for 7 and 15 months, respectively, while the co-infection of *L*. *major* and *L*. *turanica* lasted for over 25 months and, in contrast to self-healing monospecific infections, almost invariably resulted in a chronic disease [17].

The evolution in *Leishmania* appears to be driven by the opposite forces of clonality and sexual reproduction [18,19], with geographical isolation of populations (vicariance) playing an important role in any proposed model. Most of the intensely studied *Leishmania* spp. are not geographically isolated and often mixed [20–23]. *Leishmania turanica* in Central Asia presents a good and rather unique model to compare mixed and monospecific infections in natural populations. As mentioned above, in most areas of the *R*. *opimus* distribution, populations of *L*. *gerbilli*, *L*. *major*, and *L*. *turanica* are mixed with high prevalence of functionally important *L*. *major*–*L*. *turanica* co-infections [12,24]. Conversely, the populations of *L*. *turanica* in Mongolia are predominately monospecific (no documented *L*. *major* and very rare cases of *L*. *gerbilli* reported) and geographically isolated by Altai mountains [25,26].

The main goal of this work was to compare genomes of several previously characterized strains of *L*. *turanica* originated from mixed (with *L*. *major*) and monospecific geographically isolated populations in Central Asia in order to understand whether their distinct ecology is reflected at the genomic level. In addition, we aimed to determine the degree of differences between *L*. *turanica* strains from various geographic locations and compare them to those of well-studied *L*. *major*.

## Results and discussion

### Genome sequencing and variant calling in *L*. *turanica* strains

In total, eleven *Leishmania turanica* strains isolated between 1983 and 1995 from mixed and monospecific populations in Central Asia (Table 1) were sequenced at high depth using paired-end Illumina sequencing platform. All the analyzed strains were isolated from the same host species, *R*. *opimus*. Three Mongolian strains (hereafter referred as ‘MN’) and five strains from Turkmenistan (hereafter referred as ‘TM’) represent the monospecific and mixed populations, respectively. We also sequenced one strain of *L*. *turanica* from Uzbekistan and one strain of the same species from Kazakhstan to use them as an outgroup for more accurate measurement of genetic variations at species level for the analyzed groups. One of the strains (87568) turned out to be a mix of *L*. *major* and *L*. *turanica* and was excluded from further analysis.

**Table 1:** *Leishmania turanica* strains analyzed in this study.

Strain ID	Code WHO	Year of isolation	Host	Country, place (GPS coordinates) of isolation
9104	MRHO/TM/91/Marz-9104	1991	*R*.*opimus*	Turkmenistan, Teze-Yël (37°29’55"N 60°21’4"E)
9105	MRHO/TM/91/Marz-9105	1991	*R*.*opimus*	Turkmenistan, Teze-Yël (37°29’55"N 60°21’4"E)
91014	MRHO/TM/91/Marz-91014	1991	*R*.*opimus*	Turkmenistan, Teze-Yël (37°29’55"N 60°21’4"E)
9562	MRHO/TM/95/Marz-9562	1995	*R*.*opimus*	Turkmenistan, Purnuar (38°51’55"N 56°19’26"E)
9563	MRHO/TM/95/Marz-9563	1995	*R*.*opimus*	Turkmenistan, Mekan obasy (Molochniy) (37°48’14"N 58°50’34"E)
MNR1	MRHO/MN/83/Marz-MNR1	1983	*R*.*opimus*	Mongolia, Aikhingol (Jinst) (45°24’33" N 100°35’26" E)
MNR4	MRHO/MN/83/Marz-MNR4	1983	*R*.*opimus*	Mongolia, Züünbayan (44°30’8"N 110°03’12"E)
MNR14	MRHO/MN/84/Marz-MNR14	1984	*R*.*opimus*	Mongolia, Züünbayan (44°30’8"N 110°03’12"E)
KD51	MRHO/UZ/83/Marz-KD51	1983	*R*.*opimus*	Uzbekistan, Mubarek (39°14’56"N 65°08’25"E)
BK7	MRHO/KZ/87/MARZ-BK7	1987	*R*.*opimus*	Kazakhstan, Bayrkum (42°07’04"N 68°08’43"E)
87568	MRHO/UZ/87/Marz-KD87568	1987	*R*.*opimus*	Uzbekistan, Qarshi (38°51’48"N 65°47’52"E)

The MultiQC analysis for duplicate reads confirmed that all samples were sequenced to sufficient depth (average genome coverage is about 65×). Read mapping statistics and initial SNP (Single Nucleotide Polymorphism) calling statistics for sequenced samples are summarized in Table 2. Two strains, namely KD51 and BK7, possess at least two times less homozygous and more heterozygous SNPs against the reference strain LEM423 (MMEL/SU/1979/MEL) than other analyzed strains of the MN and TM groups. Notably, the number of short indels is very similar in all the strains under analysis.

**Table 2:** General mapping and SNP calling statistics for analyzed strains of *L*. *turanica*.

Strain	Mapped, %	SNPs	Insertions	Deletions	Ts/Tv	Heterozygous SNPs	Homozygous SNPs
9562	94.69	67,557	12,975	15,953	2.13	16,433	51,124
9563	93.3	67,967	13,151	15,996	2.11	16,833	51,134
9105	95.46	68,472	13,289	16,194	2.12	17,811	50,661
9104	96.54	68,557	13,038	16,096	2.12	19,523	49,034
91014	89.81	68,312	13,302	16,107	2.12	17,537	50,775
MNR1	94.87	75,769	14,030	17,391	2.11	37,946	37,823
MNR4	95.6	73,923	13,714	17,201	2.08	32,313	41,610
MNR14	96.43	73,528	13,822	17,312	2.1	31,996	41,532
BK7	96.17	83,694	14,483	17,263	2.07	58,002	25,692
KD51	95.56	83,641	14,699	17,653	2.13	57,591	26,050

Ts/Tv is transition/transversion ratio.

**Fig 1:**
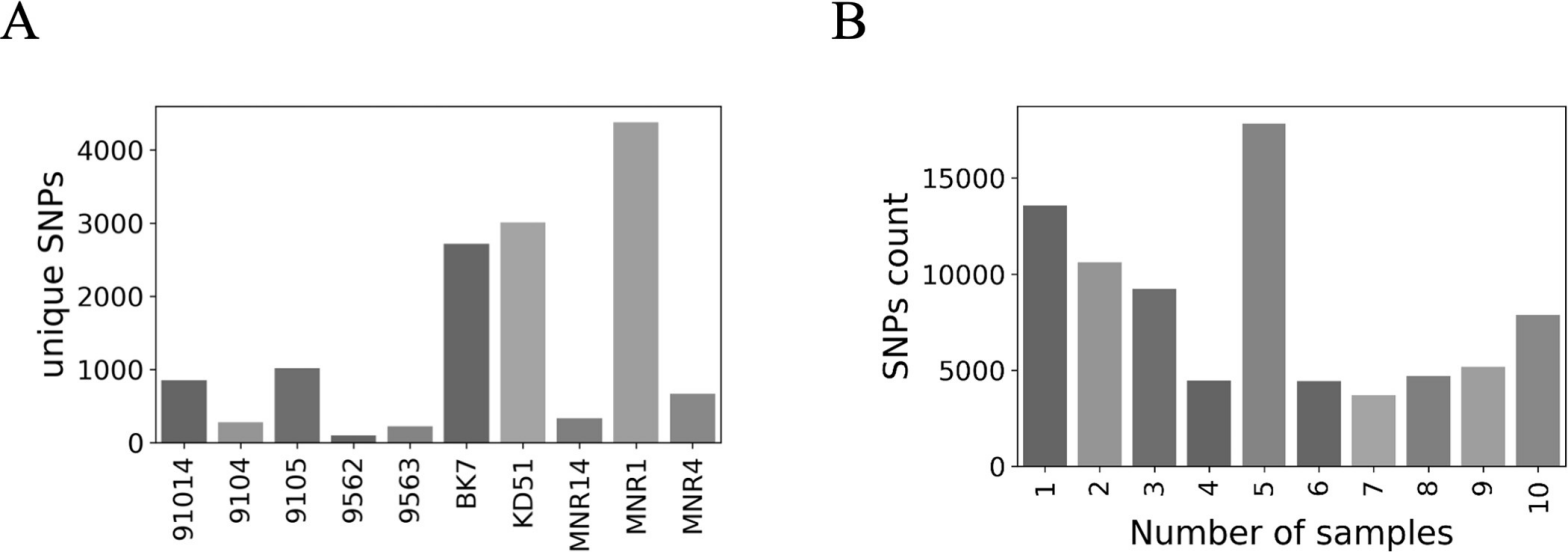
Single nucleotide polymorphisms analysis of *Leishmania turanica* strains. (A) Strain-specific SNPs. (B) Persistence of SNP in samples. The X axis shows the number of strains sharing SNPs. The shades of gray are only illustrative.

We further filtered the SNPs and produced a set 81,551 of confident homozygous SNPs in genomic positions well-covered in all sequenced samples of *L*. *turanica*. In total, about 14,000 homozygous SNPs are unique for only one strain, with MNP1 being the most divergent (about 4,000 unique SNPs) of all. Interestingly, two other MN and all TM strains are less divergent than strains from Kazakhstan and Uzbekistan (Fig 1A). Analysis of the SNP persistence among strains revealed that about 17,000 SNPs are shared by five strains (Fig 1B). Further analyses showed that this group of 5 strains share the same origin–Turkmenistan.

We also assembled the unmapped reads of all the studied strains *de novo* in order to detect genes possibly missing in the reference assembly of LEM423. We revealed 220–240 genes or gene fragments, which share common annotations, in each of the strains. This implies that the reference assembly of LEM423 [27] lacks a small portion of *L*. *turanica* genes. Among these genes, the group of surface membrane glycoprotein GP46/M-2-like genes is always present on separate small scaffolds with coverage greater than average and the number of these genes vary between strains (from 2 to 10 copies). This suggests the presence of tandemly multiplicated gene copies typical for trypanosomatid genomes and explains difficulties in assembling and annotating this locus.

### Phylogenetic inferences

**Fig 2:**
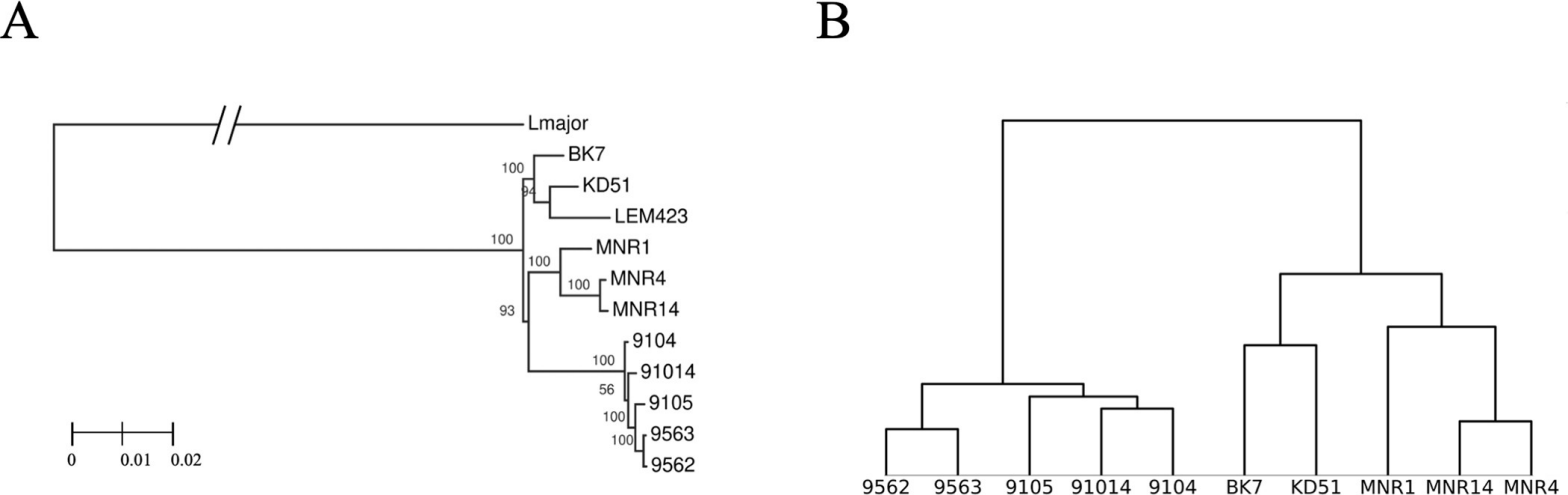
Phylogenomic analyses of *L*. *turanica* strains. (A) Maximum-likelihood phylogenomic tree of the investigated strains. *Leishmania major* is used as an outgroup. Bootstrap supports were determined using 1,000 replications. Branch length leading to *L*. *major* is manually scaled down 15× times as inter-species distance is much higher than that for strains of the same species. The scale bar represents substitutions per site. (B) Hierarchical clustering distance matrix output. All 81,551 confident homozygous SNPs were used to calculate the distance matrix.

For phylogenetic inference, we added the reference *L*. *turanica* LEM423 [27] and an outgroup species *L*. *major* Friedlin (MHOM/IL/81/Friedlin) [28]. In order to do that, we first performed orthologous genes clustering and discovered single copy orthologs between two *Leishmania* spp. Out of all genes, which included SNPs from confident set in *L*. *turanica* and had a single-copy ortholog determined in *L*. *major*, we randomly selected 2,000 genes to build a maximum-likelihood tree (Fig 2A). In agreement with previously reported data on other Trypanosomatidae spp. [29], the inter-species distance between two *Leishmania* spp. is ~20× greater than average inter-strain distance. Phylogenetic analysis confirms that MN and TM strains are grouped into separate clades. Of note, BK7, KD51, and LEM423 also grouped together (hereafter, called LEM strains), making strains originated from mixed infections paraphyletic. The external branch lengths are in concert with unique SNPs counts (Fig 1A): MNR1, KD51, and BK7 strains are furthest form their last common ancestors.

As the phylogenetic tree in Fig 2A was constructed using only a subset of SNPs, we also performed a hierarchical clustering based on all confident SNPs (Fig 2B). The results of this analysis agree with the ML-tree. Five TM strains are placed in the more secluded clade and appear even closer to each other if the distance is measured only by homozygous SNPs.

### Homozygous SNPs and codon usage in *Leishmania* spp

Next, we investigated the impact of homozygous SNPs on the codon usage in different strains of *Leishmania* sp. In all investigated strains, the general characteristics of SNPs are similar: i) about 60–65% of SNPs are intergenic; ii) on average, 4,200 genes contain at least one SNP; iii) the mean number of SNPs per gene is 2.0; and iv) the average synonymous to non-synonymous SNPs ratio is 1.2.

**Fig 3:**
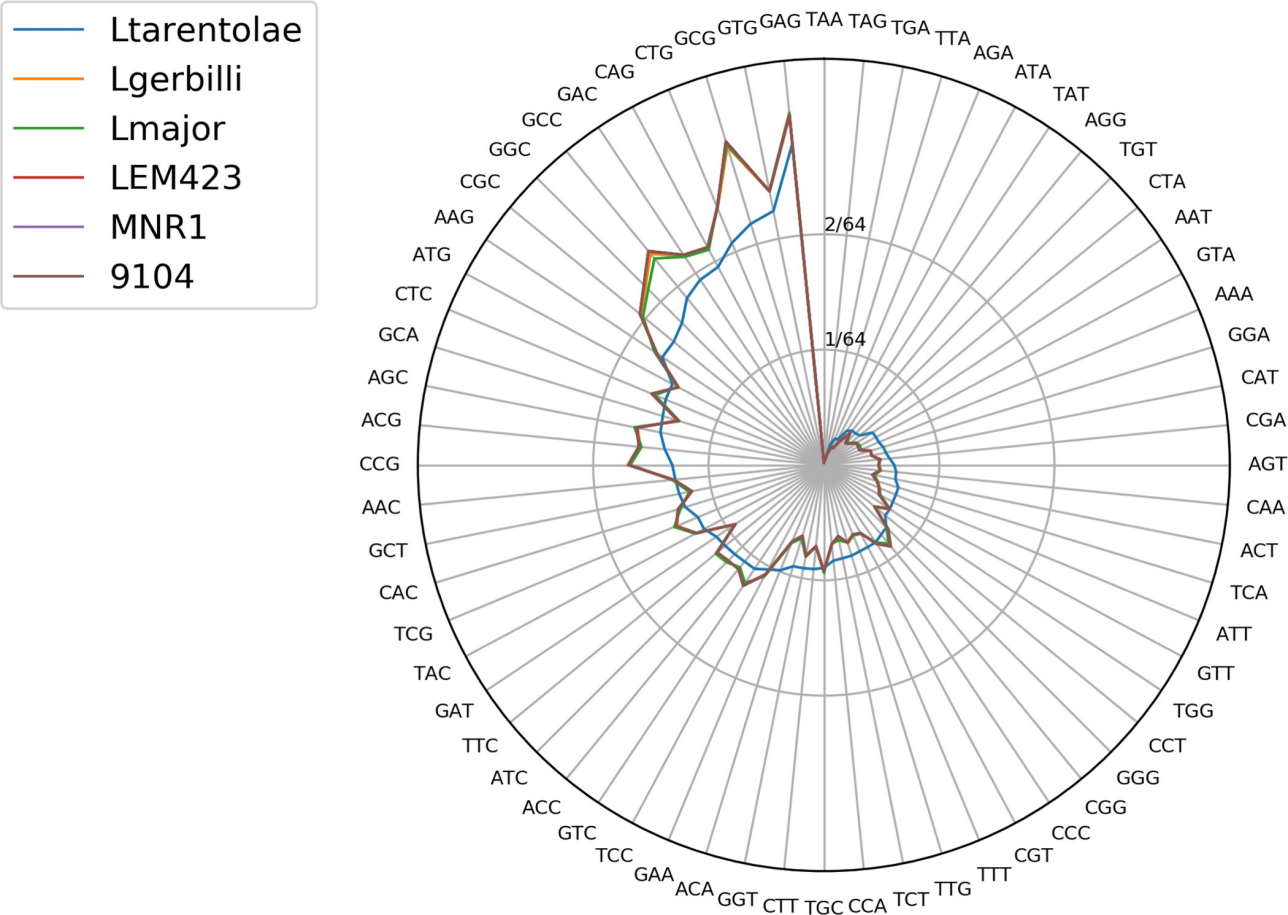
Codon usage plot. Codon frequencies for each of 64 triplets are plotted for *Leishmania tarentolae* Parrot-TarII (‘Ltarentolae’), *L*. *gerbilli* LEM452 (‘Lgerbilli’), *L*. *major* Friedlin (‘Lmajor’), *L*. *turanica* LEM423 (‘LEM423’), *L*. *turanica* MNR1 (‘MNR1’), and *L*. *turanica* 9104 (‘9104’).

Previously, we demonstrated that genomes of two *Crithidia* spp. greatly varied between strains collected in different geographic locations in terms of SNPs, yet their codon usage remained strictly species-specific [29]. We performed similar analysis for *Leishmania* spp. and demonstrated that all *L*. *turanica* strains belong to one species (the lines representing LEM423 and two other strains are fused together indicating zero overall difference in codon usage) (Fig 3). Interestingly, *L*. *gerbilli* and *L*. *major*, which are considered phylogenetically close [30], have similar codon usage pattern, but still detectable differences (for example, triplets GCC, GCG, AGC, or TCT).

### Chromosome copy number variation and genome-wide rearrangements in *L*. *turanica* strains

**Fig 4:**
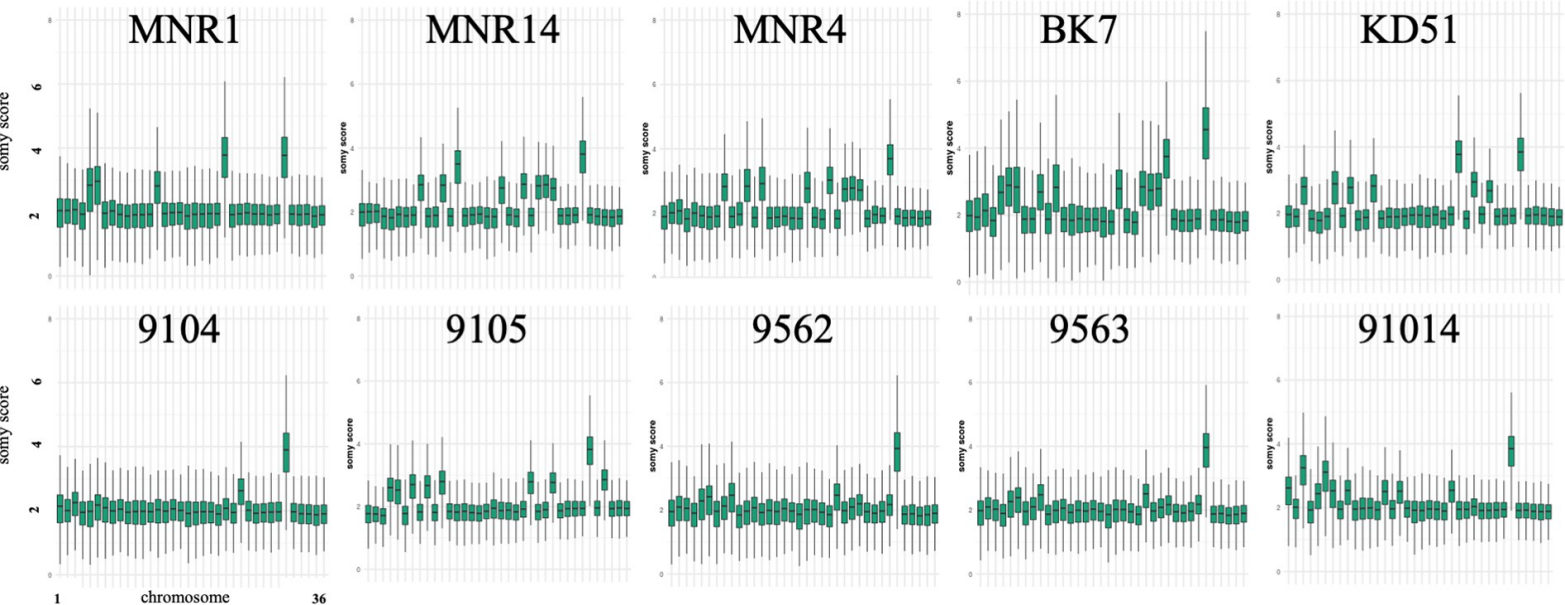
Chromosome copy number variations in sequenced strains of *L*. *turanica*.

Chromosome copy number variation (CCNV) analyzed with GIP revealed that in all analyzed strains, the chromosome 31 has a ploidy of 4, which is typical for other *Leishmania* spp. analyzed in this respect [31–33]. In particular strains, we detected either full chromosome amplifications (i.e. chromosome 23 in MNR1) or increased coverage in large regions of the chromosome (i.e. chromosomes 5 and 6 in MNR1). We did not observe specific patterns of chromosome amplification in the strains from mixed or monospecific infections; instead, we documented sporadic amplifications in different and distant strains (Fig 4). For example, chromosome 5 is amplified in strain MNR1 from Mongolia and strain 9105 from Turkmenistan, while chromosome 27 is only amplified in the strain 9104. In other *Leishmania* spp., CCNV may be even more dramatic. For example, the number of amplified chromosomes for two isolates of *L*. *mexicana* (U1103 and M379) were found to be ten and two, respectively [31]. The CCNV appears to be circumstantially linked to the recent or ongoing adaptations [34–37]. For *Leishmania* spp. with their predominantly asexual reproduction and simplified mechanisms of transcription control, aneuploidy seems to be the good compensatory mechanism for quick adaptive regulation of gene expression [18]. Of note and different from *L*. *turanica*, its close phylogenetic relative *L*. *major* does not display such a variability in chromosome copy number.

**Fig 5:**
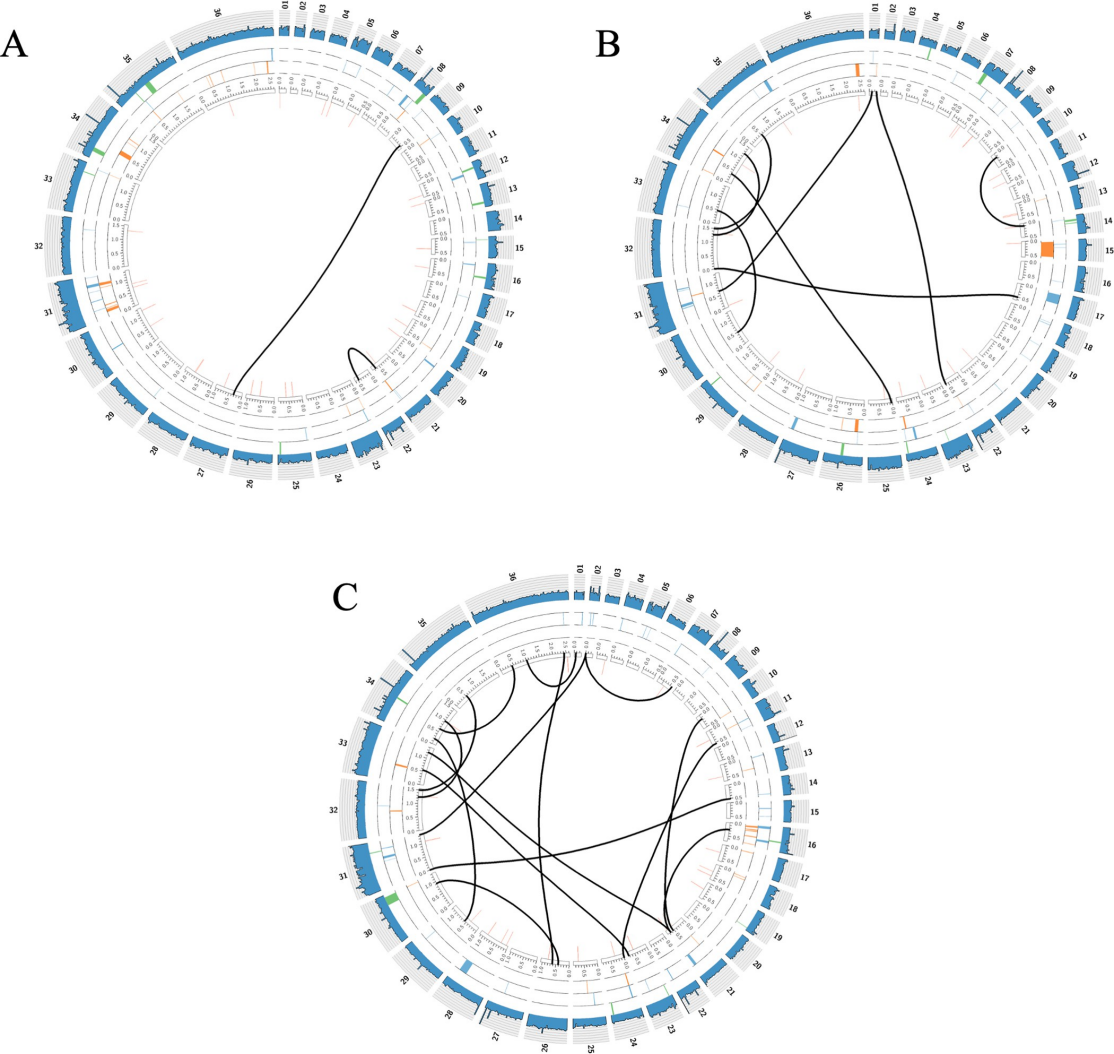
Whole-genome-scale structural variations specific for *L*. *turanica* strains. (A) Single infections MN strains (coverage track exemplified by MNR4). (B) Mixed infections LEM strains (coverage track exemplified by KD51). (C) Mixed infections TM strains (coverage track exemplified by 9105). Outer histograms (blue) show chromosome read coverage, inner tracks with highlights show the inversions (green), deletions (blue), duplications (orange), and insertions (red). Black lines show inter-chromosome translocations.

Genome rearrangements of other types, including large tandem duplications, large deletions, inversions or translocations between chromosomes were investigated with the DELLY tool. We detected series of translocations, in which chromosome 8 was involved. These translocations persist in all analyzed strains and involve repeat and telomeric/sub-telomeric regions of chromosomes, therefore, we excluded them from further analysis, as they might be caused by repetitive loci read multimapping artifacts. For other types of large-scale variations, we identified the events common to specific clades in Fig 2. These variations are presented in Fig 5 for representatives of the LEM (mixed), MN (monospecific), and TM (mixed) groups. We conclude that at the level of large-scale genomic variations these groups have different evolutionary patterns and different chromosomes appear to be involved in these events. For example, translocations (black lines) are more common for the strains from mixed infections of both LEM (9 translocations) and TM (35 translocations) groups than for their MN single-infection kin (2 translocations).

**Fig 6:**
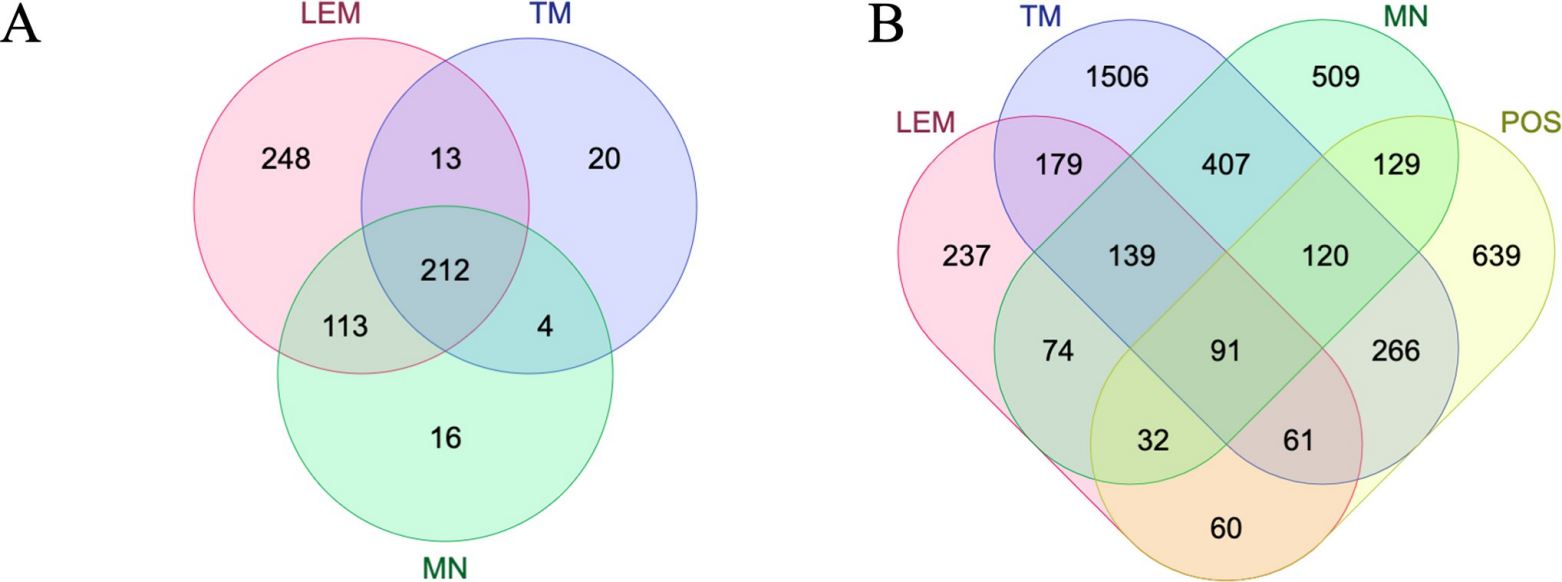
Venn diagrams showing the intersections of the gene sets under investigation. (A) Intersection between amplified (> 2n) genes for MN, LEM, and TM groups. (B) Intersection between sets of genes with confident homozygous SNPs and genes under positive selection (POS) for MN, LEM, and TM groups. Genes IDs are provided after assembly of *L*. *turanica* LEM423.

To understand the possible evolutionary impact of described genome variations, we determined, what genes are involved in these changes (S1 Table). For this, we analyzed sets of amplified genes (present in more than 2 copies relative to the diploid LEM423 genome) and genes with confident homozygous SNPs (Tables 2 and S1) in detail. Five hundred eighty-six, 345, and 249 genes were documented amplified for LEM, MN, and TM strains, respectively. Out of these, 212 genes (out of 626 in total) were amplified in all three groups, and large proportions of genes were amplified in two groups (Fig 6A). Similarly, we analyzed the genes that were significantly depleted with reads (gene deletions), and found very few such cases (less than 8 genes per strain). Therefore, we conclude that gene duplication events are prevalent in evolution of *Leishmania* genomes. Next, we compared sets of genes with confident homozygous SNPs and found that a significantly smaller proportion of them (230 out of 4,449 in total) is shared by the MN, LEM, and TM groups (Fig 6B). Out of these, only 2 genes were commonly shared by all three groups in categories amplified and confident homozygous SNPs-containing. The combination of the following two factors may explain the observed effect: i) the amplified copies remain under strong selection pressure and do not multiply SNPs, because each copy defines precise gene dosage [38,39], and/or ii) the amplification is recent.

Finally, we analyzed a set of positively selected genes in *L*. *turanica* strains (1,398 genes in total) and overlapped this dataset with that for genes with confident homozygous SNPs (Fig 6B). There was no clear prevalence of strain-specific selection acting on the group of genes in any strain, 91 genes out of 230 shared by all groups were under positive selection. We also conducted Gene Ontology enrichment studies for the genes under positive selection and found no obvious functional enrichment. To assess the question of selection on the branches leading to MN and TM clades, we used branch models from PAML and found 19 and 46 genes under positive selection on these branches, respectively. These sets have no overlap.

### Conclusions

Taken together, our results illustrate that evolutionary differences between strains of *L*. *turanica* originated from mixed and monospecific infections are not dramatic in terms of codon usage (Fig 3) or CCNV (Fig 4). Instead, we documented very targeted evolutionary changes, focused on groups of genes (S1 Table and Fig 6). Conversely, on the level of large-scale genomic rearrangements, we confirmed that different genomic loci and different types of rearrangements were implicated in groups of species under analysis (for example, strains from monospecific infections have fewer translocation events than their counterparts from mixed infections, Fig 5). We conclude that large-scale genomic rearrangements may precede “fine tuning” of the speciation process in *Leishmania turanica*.

## Materials and methods

### Sample collection, DNA preparation, and whole-genome sequencing

Strains of *Leishmania turanica* from the collection of Martsinovsky Institute of Medical Parasitology, (Table 1) were cultivated as described previously [40,41]. These strains were isolated between 1983 and 1995 in Kazakhstan, Mongolia, Turkmenistan, and Uzbekistan from great gerbils (*Rhombomys opimus*). The strain identity was confirmed as in [42]. Total genomic DNA was isolated from 10 ml of the log-phase cultures using DNeasy Blood & Tissue Kit (Qiagen, Hilden, Germany) according to the manufacturer’s instructions. Samples were sequenced using HiSeq 4000 platform (Illumina, San Diego, USA) in PE150 mode at SkolTech Genomics core facility (Moscow, Russia) to the depth of approximately 7 million read pairs per sample, giving mean *Leishmania* genome coverage of 65×. The obtained data were deposited to GenBank (BioProject PRJNA888552). One of the strains (87568) was a mix of *L*. *major* and *L*. *turanica* and was excluded from further analysis. In total, our dataset included 3 strains from Mongolia (labeled MN), 5 strains from Turkmenistan (labeled TM), and 3 strains (from Uzbekistan and Kazakhstan, 1 reference, 2 sequenced in the frame of this work) used as an outgroup.

### Read mapping and processing

The paired-end Illumina reads of *L*. *turanica* strains were trimmed for quality and sequencing adaptors with Trimmomatic v. 0.36 [43] and quality controlled using FastQC v. 0.11.8 [44] and MultiQC v. 1.13 [45]. Each sample was mapped to the reference genome of *L*. *turanica* LEM423 [27] using the BWA MEM v. 0.7.17 [46]. Read processing was done with SAMtools v. 1.9 [47] using ‘fixmate’, ‘view’, ‘sort’ and ‘markdup’ commands. Sorted and deduplicated bam files were used for all further processing.

### *De novo* assembly of unmapped reads

Reads, not mapped on the nuclear genome, were collected with SAMtools using a custom Python script (read pair considered unmapped if any read of the pair was unmapped). Unmapped read pairs of each strain were assembled *de novo* with SPAdes v. 3.13.0 with default settings [48]. Genes on assembled scaffolds were predicted with Augustus v. 3.3.2 [49] with species model for *Leishmania tarentolae*. Predicted proteins were BLASTed against the local copy of UniProt database [50] using BLASTp.

### Variant calling and processing

Initial variant calling was done with Freebayes v. 0.9.21 [51] with the following options "—read-indel-limit 1—read-mismatch-limit 3—read-snp-limit 3—min-alternate-fraction 0.05—min-base-quality 10—min-alternate-count 2—pooled-continuous -p 2”. Collected variants were filtered using custom Python script, filtering was done on all analyzed samples simultaneously. Only homozygous SNPs (single nucleotide polymorphisms) with coverage of at least 15 reads in all sequenced samples and with calling quality over 20 were gathered for further analyzes. These SNPs were inserted into the genome sequence to obtain strain-specific gene sequences using VCF-consensus tool from VCFtools v. 0.1.16 [52]. Allele frequency histogram and allele depth of each SNP were analyzed with a custom Python script and plotted using seaborn Python library.

### Analysis of gene orthologs

Orthologous gene sets between *L*. *major* and *L*. *turanica* were determined by OrthoFinder v. 2.5.4 [53] on annotated protein-coding genes of *L*. *major* strain Friedlin and *L*. *turanica* strain LEM423. Single-copy orthologs were used to determine 1-to-1 correspondence between genes of two species. Sets of *L*. *turanica* genes not included into an orthologous group with any *L*. *major* gene were considered *L*. *turanica*-specific.

### Phylogenetic analysis

Gene sequences of *L*. *major* Friedlin [31] were used for an outgroup. A set of confident homozygous SNPs determined in *L*. *turanica* strains and a set of 1-to-1 orthologs between two *Leishmania* spp. were used to randomly select 2,000 genes for multiple sequence alignment. Each selected gene included at least one SNP from the confident SNPs set. The sequence alignments were done with MAFFT v. 7.475 [54] and combined into a single multiple sequence alignment file with custom Python script. The tree was inferred in RAxML-NG v. 1.0.2 program [55] with 1,000 bootstrap replicates under GTR + G model and visualized with Toytree Python library [56].

### Gene variation and selection

Group-specific SNPs were extracted from the set of confident SNPs using a custom Python script. Genes with group-specific SNPs were extracted using the combination of BEDtools v. 2.30.0 [57] ‘intersect’ and ‘getfasta’ programs. General SNP effect statistics was collected using SnpEff v. 5.1 [58].

The site and branch tests (CODEML program from the PAML package v. 4.9 [59]) were used to test for selection. Positively-selected sites were chosen by comparing the likelihood of the M7/M8 models for each gene. Branch tests were performed for i) a branch leading to the common ancestor of MNR1, MNR4, and MNR14 as a foreground branch or ii) a branch leading to the common ancestor of 9104, 9105, 91014, 9562, and 9563 strains as a foreground branch to find the genes under positive selection during evolution of Mongolian and Turkmen strains, respectively. All other branches were considered a background branches, free-ratio, one-ratio, and two-ratios models were compared with the likelihood-ratio test, LRT.

### Genome-level structural variations analysis

The structural variations on the whole-genome level, including translocations, long inversions, tandem duplications, long deletions, and locus copy number variations (including both gene and chromosome copy number variations) were detected with the GIP pipeline v. 1.0.9 [60]. The output of the DELLY v. 1.1.5 [61] was re-analyzed with a custom Python script to detect specific variations in MN and TM groups. Data were visualized using modified scripts from the GIP pipeline.

## Supporting information

S1 Table Datasets of amplified genes and genes with confidents homozygous SNPs in analyzed groups of *L*. *turanica*. (XLSX)
